# Sustained antibacterial coating with graphene oxide ultrathin film combined with cationic surface-active agents in a wet environment

**DOI:** 10.1038/s41598-022-21205-4

**Published:** 2022-10-18

**Authors:** Hirofumi Miyaji, Yukimi Kanemoto, Asako Hamamoto, Kanako Shitomi, Erika Nishida, Akihito Kato, Tsutomu Sugaya, Saori Tanaka, Natsuha Aikawa, Hideya Kawasaki, Syun Gohda, Hironobu Ono

**Affiliations:** 1 Department of Periodontology and Endodontology, Faculty of Dental Medicine, Hokkaido University, N13W7, Kita-ku, Sapporo, Hokkaido 060-8586 Japan; 2 Division of Periodontology and Endodontology, Department of Oral Rehabilitation School of Dentistry, Health Sciences University of Hokkaido, 1757 Kanazawa, Tobetsu-cho, Ishikari-gun, Hokkaido 061-0293 Japan; 3 Division of General Dentistry Center for Dental Clinics, Hokkaido University Hospital, N14W5, Kita-ku, Sapporo, Hokkaido 060-8648 Japan; 4 Department of Chemistry and Materials Engineering, Faculty of Chemistry, Materials and Bioengineering, Kansai University, 3-3-35 Yamate-cho, Suita, Osaka 564-8689 Japan; 5 Nippon Shokubai Co., Ltd, 5-8 Nishiotabi-cho, Suita, Osaka 564-0034 Japan

**Keywords:** Infection control in dentistry, Graphene, Drug delivery

## Abstract

Antimicrobial surfactants contained in mouthrinse have excellent efficacy, but are not retained on the tooth surface (are rinsed away) due to their low water resistance and thus do not exhibit sustained antibacterial activity. We have developed a new coating method using graphene oxide (GO) that retains the surfactant on the tooth surface even after rinsing with water, thus providing a sustained antibacterial effect. Ultra-thin films of GO and an antimicrobial agent were prepared by (1) applying GO to the substrate surface, drying, and thoroughly rinsing with water to remove excess GO to form an ultrathin film (almost a monolayer, transparent) on the substrate surface, then (2) applying antimicrobial cationic surface active agents (CSAAs) on the GO film to form a composite coating film (GO/CSAA). GO/CSAA formation was verified by scanning electron microscopy, Raman spectroscopy, X-ray photoelectron spectroscopy, and ζ-potential and contact angle measurements. GO/CSAA was effective at inhibiting the growth of oral pathogens for up to 7 days of storage in water, and antibacterial activity was recovered by reapplication of the CSAA. Antibacterial GO/CSAA films were also formed on a tooth substrate. The results suggest that GO/CSAA coatings are effective in preventing oral infections.

## Introduction

Reducing the number of tooth extractions required due to diseases such as dental caries and periodontitis will help to extend longevity and increase quality of life^1,2^, but maintaining daily oral care using a toothbrush is often difficult for elderly people due to a decline in physical and cognitive functions^3^. Various mouthrinse agents have been used to aid in oral care, but although alcohols, hypochlorite, surfactants, and various organic compounds in mouthrinses have high antimicrobial activity, they quickly volatilize or are not water-resistant and thus are rinsed away by water. This makes it difficult to retain antimicrobial compounds on tooth surfaces, requiring daily use of mouthrinses^4,5^. There is therefore need for a new antimicrobial coating method that remains effective, even in a wet environment such as the mouth.

Graphene oxide (GO) is a nanocarbon material composed of sheets with a large aspect ratio (ratio of width to thickness); the sheets are approximately 1 nm thick and several micrometers wide^6^. GO is prepared by the oxidation of graphite with a strong oxidant such as potassium permanganate in sulfuric acid^7^. GO sheets contain various oxygen-containing functional groups, such as epoxy, hydroxyl, and carboxylic acid groups^8^. The shape of GO and its large number of oxygen functional groups allows GO to strongly interact with a variety of molecules and polymers, resulting in good adhesion to various substrates. Using this property, we speculated that the combination of antimicrobial agents with GO could be used to create water-resistant antimicrobial coatings with long-term stability on substrate surfaces.

Our strategy for combining GO with antimicrobial agents was as follows. An ultrathin GO film (almost a monolayer) was first formed on the substrate surface. The film was transparent and did not affect the color of the substrate. Quaternary amines, which have antibacterial and antiviral activities^9–11^, were then bound as cationic surface active agents (CSAAs) to the anionic GO film to form a composite. Normally, when anionic GO is combined with a cationic substance, GO readily aggregates and no longer disperses^12^, however, the strong adhesion of GO to the substrate prevents this. In the present study, we characterized GO films combined with several types of CSAAs and assessed their antibacterial properties. Our findings show that GO and CSAA composite film coatings exhibit antibacterial activity, even after rinsing with water.

## Materials and methods

### Preparation of GO dispersion

The GO dispersion was synthesized as follows. Graphite (15 g, FUJIFILM Wako Pure Chemicals Co., Ltd., Osaka, Japan) and 640 g of sulfuric acid (FUJIFILM Wako Pure Chemicals Co., Ltd.) were mixed, and then 45 g of potassium permanganate (FUJIFILM Wako Pure Chemicals Co., Ltd.) was added at room temperature. The mixture was allowed to react for 2 h, and then 1070 mL of water and 42 mL of 30% aqueous hydrogen peroxide (FUJIFILM Wako Pure Chemicals Co., Ltd.) were added to stop the reaction. The product was purified by static sedimentation to remove the supernatant and repeated redispersion in ion-exchanged water. After purification, the reaction solution was homogenized using a homogenizer (10,000 rpm, HG-200, AS ONE Corporation, Osaka, Japan) to obtain the GO dispersion.

### Preparation of GO ultrathin film coating combined with CSAA

A fused quartz polishing plate (20 mm × 20 mm × 1 mm, AS ONE Corporation) was heated at 700 °C for 2 h under an air atmosphere to burn off surface organic matter. The concentration of the GO dispersion was adjusted to 0.1%, and 0.4 mL of the GO dispersion was drop-cast on the heat-treated quartz substrate to completely cover the quartz substrate. After drying under vacuum at room temperature, the substrate was vigorously sprayed with water to remove excess GO, and then vacuum dried again.

Benzalkonium chloride (BAC, FUJIFILM Wako Pure Chemicals Co., Ltd.), cetylpyridinium chloride (CPC, Tokyo Chemical Industry Co., Ltd., Tokyo, Japan), and benzethonium chloride (BZC, Tokyo Chemical Industry Co., Ltd.) were used as CSAAs. Each CSAA was prepared as a 0.1% aqueous solution, and the GO-coated quartz substrate was immersed in each solution for 10 s. The substrate was then immediately rinsed thoroughly with water and vacuum dried to obtain a GO ultrathin film coating combined with CSAA (denoted as GO/CSAA, e.g., GO/BAC, GO/CPC and GO/BZC). For comparison purposes, we prepared an untreated quartz substrate, a substrate with a GO coating only, and substrates immersed only in each CSAA solution.

### Characterization of GO/CSAA

Each quartz substrate was investigated by Raman spectroscopy (excitation wavelength 532 nm, NSR-3100, JASCO Corporation, Hachioji, Japan) to detect the GO film. In addition, the morphology of the GO film with/without CSAA on the quartz substrates was observed by field emission scanning electron microscopy (SEM, JSM-7600F, JEOL Ltd., Akishima, Japan). The ratio of the GO-coated area to the total substrate area was calculated using image analysis software (ImageJ, ver. 1.41, National Institutes of Health, Bethesda, MD, USA).

To measure the transmittance of the GO film, the absorbance at 660 nm for untreated, GO alone, CSAA alone, and GO/CSAA treated substrates was measured using a UV–Visible spectrophotometer (UV-3100, Shimadzu Corporation, Kyoto, Japan). The transmittance of each substrate was calculated by setting the transmittance of the untreated quartz substrate as 100%.

The elemental composition and chemical states of the materials on each quartz substrate were investigated by X-ray photoelectron spectroscopy (XPS, Al-Kα, AXIS-NOVA, Shimadzu Corporation). The C1s, N1s, O1s, and Cl2p XPS spectra were then analyzed to detect the presence of GO and CSAA. C1s spectra show the bonding states of GO, such as C–C, C–O, and CO–O– bonds, and N1s spectra show the quaternary amine structure of the CSAA.

ζ-Potential measurements of GO solutions with different benzyldodecyldimethylammonium chloride (BAC_12_) concentrations were performed at 25 °C using a Zetasizer Nano ZSP (Malvern Panalytical, Ltd., Malvern, UK). The average value of six measurements was estimated for the ζ-potential measurements.

The hydrophilicity of each quartz substrate was investigated by the sessile drop method using a contact angle meter (DMs-200; Kyowa Electronic Instruments Co., Ltd., Tokyo, Japan). To confirm whether the ultrathin film formed on different substrate surfaces, the contact angles on a polyethylene terephthalate (PET) film (T-60, 50 μm, Toray Industries, Inc., Tokyo, Japan) and a polystyrene dish (tissue culture dish, TPP Techno Plastic Products AG, Trasadingen, Switzerland) were measured in the same way. In addition, to evaluate the stability of GO/CSAA in water, GO/CSAA-treated quartz substrates were stored in 100 mL of water for 7 or 14 days, the substrates were removed from the water, dried, and the contact angles were measured.

### Antibacterial effect of GO/CSAA

The same procedure used for the quartz substrate was employed to coat the bottom of a 48-well culture plate with GO/CSAA. A GO dispersion (100 µL, 0.1%) was added (drop-casting) to each of the 48 wells to completely cover the bottom of the well. After drying at room temperature, the bottom of each well was vigorously sprayed with air–water to remove excess GO using a dental spray gun (J. Morita. Corp., Tokyo, Japan), and then dried. Next, 100 µL of 0.1% CSAA (BAC, CPC or BZC) was dropped into the GO-coated wells. After rinsing with water three times and drying, we obtained GO/CSAA on the bottoms of the 48 wells and compared their antibacterial properties to untreated, only GO-coated, and only CSAA-treated wells.

We prepared bacterial suspensions of two oral pathogens^13,14^. Suspension (1) contained *Streptococcus mutans* (ATCC 35668) (1.0 × 10^7^ CFU/mL) in culture medium (brain heart infusion medium, Eiken Chemical Co., Ltd., Tokyo, Japan) with antibiotics (0.1% gramicidin (FUJIFILM Wako Pure Chemicals Co., Ltd.), 0.1% bacitracin (FUJIFILM Wako Pure Chemicals Co., Ltd.), and 1% sucrose (FUJIFILM Wako Pure Chemicals Co., Ltd.). Suspension (2) contained *Actinomyces naeslundii* (ATCC 27039) (1.0 × 10^7^ CFU/mL) in culture medium (Actinomyces broth, Becton Dickinson and Company, Franklin Lakes, NJ, USA). Each suspension was added to individual wells. After anaerobic incubation at 37 °C for 24 h, the turbidity of each culture medium was measured at 590 nm using a colorimeter (CO7500 Colourwave, Funakoshi Co., Ltd., Tokyo, Japan). To detect bacterial growth, the relative turbidity was calculated based on the turbidity of an untreated well.

We also conducted comparable testing of GO ultrathin and thick films. A GO dispersion (100 µL of 0.1%) was dried in a well and not treated with water spray to prepare the thick GO film. Turbidity tests were conducted for untreated, GO ultrathin film-treated and GO thick film-treated wells to compare their antibacterial activities in the same manner as described above.

### Antibacterial persistence of GO/CSAA during water storage

To evaluate the sustained antibacterial effect of each preparation in water, GO/CSAA-treated 48-well plates were stored in 5 L of water for 1, 3 and 7 days before antibacterial evaluation. The plates were removed from the water and dried, and a bacterial suspension (prepared as described above) was then added to each well. After 24 h of anaerobic incubation at 37 °C, the relative turbidity of each culture medium was determined based on the turbidity of an untreated well (not stored in water).

GO/BAC-treated 48-well plates were also kept in 5 L of water for 28 days, then incubated with the bacteria as described above, and the turbidity was then measured after 24 h of incubation and compared with untreated and GO/BAC-treated wells (both not stored in water). In addition, to investigate the CSAA rebinding activity of GO ultrathin films after long-term storage in water, 0.1% BAC was dropped again (reprocessing) on the GO/BAC-treated wells already stored in water for 28 days. After rinsing with water three times and drying, the wells were inoculated with *S. mutans* or *A. naeslundii* in culture medium and the relative turbidity after 24 h of incubation was calculated.

### Antibacterial effect of GO/CSAA coating on human teeth

Human tooth substrates were prepared from teeth provided by patients attending Hokkaido University Hospital who had provided informed consent. The teeth were extracted as part of routine treatment. The protocol for the clinical study was reviewed and approved by the Hokkaido University Hospital Institutional Review Board for Clinical Research (Approval No. 17–222). Experiments on human teeth were carried out in accordance with the relevant guidelines and regulations.

To investigate the clinical effectiveness of GO/CSAA, the GO ultrathin film coating of tooth surfaces (prepared by modifying the method used for the quartz substrate) was combined with CPC, which is clinically used as a mouthrinse agent^15^. This allowed CPC to be retained on the tooth surfaces and act as an antibacterial agent. After trimming and ultrasonic cleaning, a human tooth (dentin) block substrate was immersed in 0.01% GO dispersion for 10 s for surface coating and immediately dried with high-pressure air. The tooth substrate was then immersed in 0.1% CPC solution and immediately rinsed thoroughly with water. Untreated, GO-treated only, and CPC-treated only tooth substrates were prepared for comparison.

The surface of each tooth substrate was evaluated by XPS elemental analysis (for C1s, Ca2p, N1s, Na1s, O1s, and P2p) and Raman spectroscopy, and then the antibacterial activity of each tooth substrate was evaluated by seeding with *S. mutans* (using the same culture conditions as described above). Viable bacteria adhering and proliferating on each dentin substrate were fluorescently stained using a LIVE/DEAD BacLight Bacterial Viability kit (Thermo Fisher Scientific, Waltham, MA, USA), and the region stained by live cells was quantified using a fluorescence microscope (BZ-9000 BioRevo, Keyence Corporation, Osaka, Japan) and ImageJ analysis software.

### Cytocompatibility of GO/BAC

GO/BAC was prepared on glass-based dishes (AGC Techno Glass Co. Ltd., Haibara, Japan) for fluorescence staining, and the bottom of a 96-well culture plate (for cell viability and cytotoxicity assays) by the same method used for the turbidity assessments (with slight modification of the GO and BAC liquid volume). Fibroblastic NIH3T3 cells (1 × 10^4^, RIKEN BioResource Center, Tsukuba, Japan) and culture medium (MEM alpha, GlutaMAX-I; Thermo Fisher Scientific) supplemented with 10% fetal bovine serum (Qualified FBS; Thermo Fisher Scientific) and 1% antibiotics (penicillin–streptomycin; Thermo Fisher Scientific) were inoculated on each substrate to examine its biocompatibility properties. After 24 h of incubation at 37 °C in a 5% CO_2_ environment, the cultured cells were fluorescently stained using a LIVE/DEAD Viability/Cytotoxicity kit for mammalian cells (Thermo Fisher Scientific) and observed using fluorescence microscopy. Furthermore, cell viability and cytotoxicity were examined using a water-soluble tetrazolium salt (WST)-8 assay kit (Cell Counting Kit-8, Dojindo Laboratories, Mashiki, Japan) and a lactate dehydrogenase (LDH) assay kit (Cytotoxicity LDH Assay Kit-WST, Dojindo Laboratories), respectively. The absorbance at 450 nm (WST-8 activity) or 490 nm (LDH activity) was measured using a microplate reader (Multiskan FC, Thermo Fisher Scientific).

### Statistical analysis

Quantitative parameters were calculated as mean + standard deviation. A two-tailed one-way analysis of variance with Tukey’s HSD post-hoc test was used to determine statistical significance, with P < 0.05 being considered significantly different. The analysis was performed using the SPSS software package (version 11.0; IBM Corporation, Armonk, NY, USA).

## Results and discussion

### Characterization of GO/CSAA

**Figure 1:**
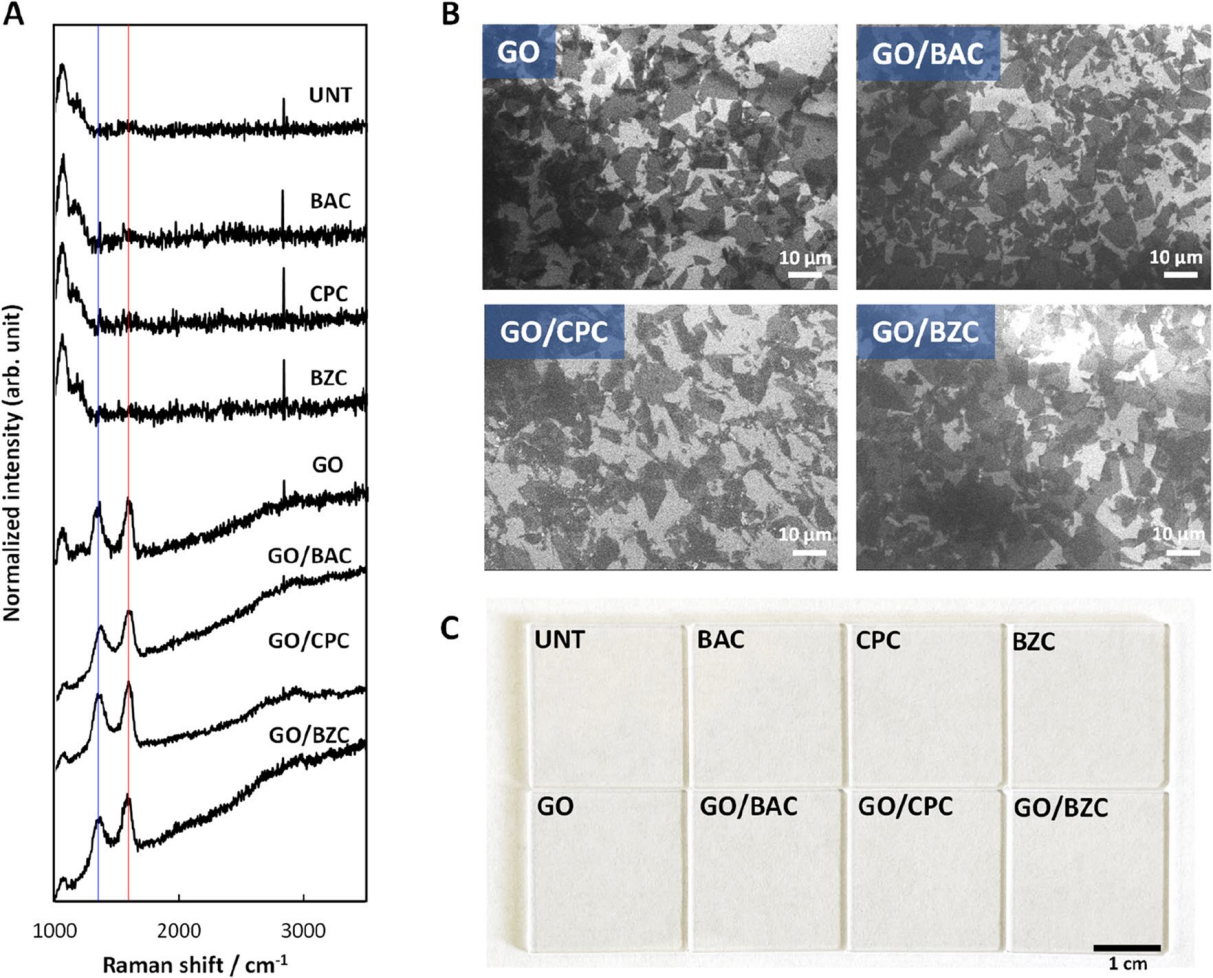
Characterization of GO/CSAA on quartz substrate. (**A**) Raman spectroscopy analyses. The red and blue lines indicate 1600 cm^-1^ and 1350 cm^-1^ (G and D bands), respectively. (**B**) SEM images of quartz substrates. (**C**) Digital photographs of quartz substrates. *BAC* benzalkonium chloride, *BZC* benzethonium chloride, *CPC* cetylpyridinium chloride, *CSAA* cationic surface active agent, *GO* graphene oxide, *SEM* scanning electron microscopy, *UNT* untreated.

Raman spectral analysis (Fig. 1A) of the surface of quartz substrates treated with GO alone and GO/CSAA showed GO-derived peaks at 1600 cm^−1^ and 1350 cm^−1^ (G and D bands, red and blue lines in Fig. 1A, respectively), suggesting the presence of GO on the surface of the substrate. The peak observed at around 2800 cm^−1^ is due to instrument noise. SEM images of the surface of the quartz substrate treated with GO only or GO/CSAA (Fig. 1B) showed a GO ultrathin film coating composed of a single to several layers of GO^16^. Since all of the substrates had been rinsed with water, GO appeared to have adhered to the substrate surface and was not removed by rinsing with water. The average ratio of GO film coverage to the substrate surface was calculated from the SEM images as 80.8% for GO and 75.9% for GO/CSAA, suggesting that secondary CSAA treatment and rinsing with water removes little GO from the substrate.

Digital photographs of GO- or GO/CSAA-treated quartz substrates are shown in Fig. 1C. The transmittance for all substrates was more than 99% (above the detection limit), using the untreated substrate as 100%, indicating that the GO ultrathin film coating is almost transparent and does not affect the color of the substrate.

**Figure 2:**
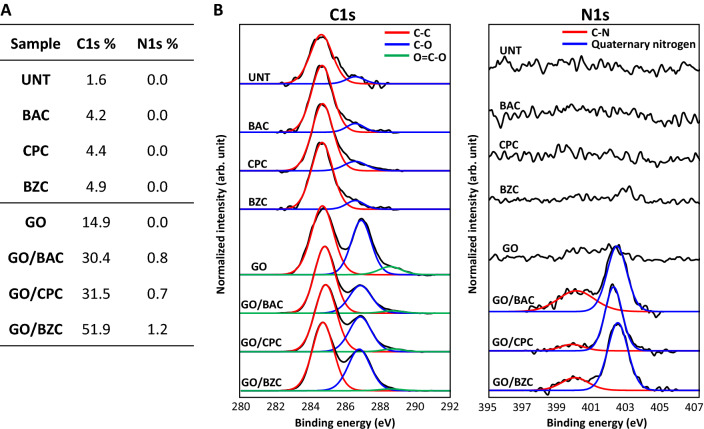
XPS analysis of GO/CSAA on quartz substrate. (**A**) C and N content. (**B**) XPS spectral analysis of C1s and N1s. *BAC* benzalkonium chloride, *BZC* benzethonium chloride, *CPC* cetylpyridinium chloride, *CSAA* cationic surface active agent, *GO* graphene oxide, *XPS* X-ray photoelectron spectroscopy, *UNT* untreated.

Figure 2A shows the XPS elemental analysis results for each substrate surface. The C content increased for the CSAA-only treated substrate surfaces compared to the untreated surface, and no N (contained in quaternary amines) was detected. This indicates very little residual CSAA on the substrate surface (without the GO ultrathin film) after treating with CSAA alone, and thus most of the CSAA was removed by the water rinsing process. GO treatment alone increased the C content, presumably due to residual GO on the substrate surface after water spraying. This is supported by the C1s and N1s XPS spectra of each substrate surface (Fig. 2B); GO- and GO/CSAA-treated substrates consistently showed GO bonding states, such as C–C, C–O, and CO–O– bonds^8^, which is consistent with the results of the Raman analysis and SEM observations of residual GO.

CSAA treatment following GO ultrathin film formation increased the content of both C and N compared to GO treatment alone (Fig. 2A). The C1s and N1s XPS spectra showed that the intensity of the C–O peak decreased in GO/CSAA compared to GO alone, and the quaternary amine structure of CSAA was detected in GO/CSAA (Fig. 2B). These results suggest that CSAA is retained on the GO film on the substrate surface even after the water rinsing process. In addition, no Cl (the counter anion of CSAA) was detected on any of the substrates (Supplementary Fig. 1, left), suggesting that ion exchange occurs between the CSAA and GO to complex the CSAA with the GO film. We do not discuss the O content and O1s XPS spectra (Supplementary Fig. 1, right) because of the significant influence of the quartz substrate.

**Figure 3:**
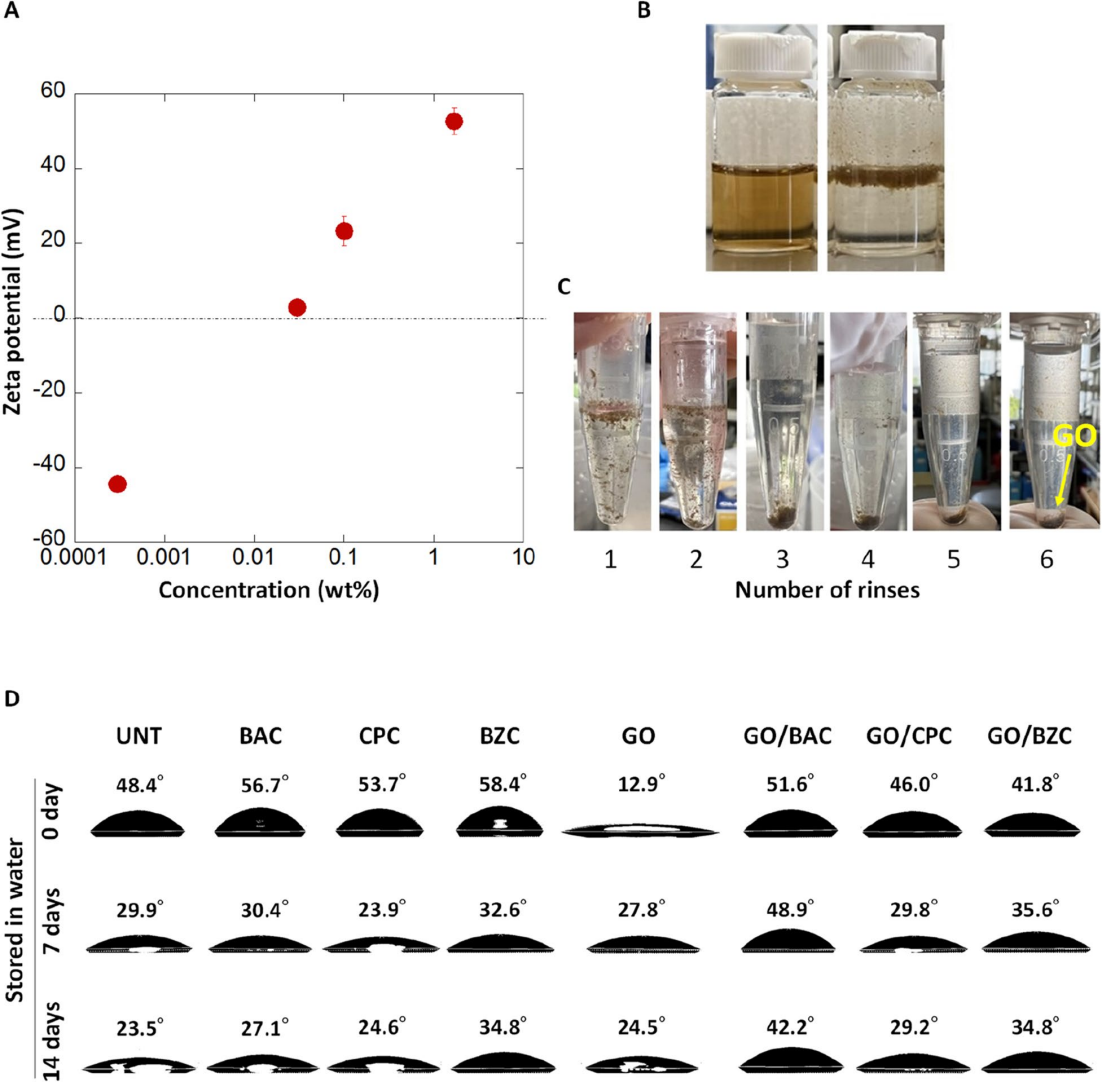
ζ-Potential of GO/BAC_12_ and hydrophilicity of GO/CSAA on quartz substrate. (**A**) ζ-Potential of GO dispersion at various BAC_12_ concentrations. (**B**) Photographs of GO dispersions with different concentrations of BAC_12_, left to right: 0.0003 and 0.03 wt%. (**C**) Photographs of GO dispersion after repeating water rinsing. (**D**) Contact angle measurements. The number above each droplet is the contact angle for the sample. *BAC* benzalkonium chloride, *BAC*_*12*_ benzyldodecyldimethylammonium chloride, *BZC* benzethonium chloride, *CPC* cetylpyridinium chloride, *CSAA* cationic surface active agent, *GO* graphene oxide, *UNT* untreated.

ζ-Potential measurements are among the most effective methods for determining surfactant absorption on a GO surface^17^. The ζ-potential for a GO solution in the presence of BAC_12_ was measured as a function of the BAC_12_ concentration (Fig. 3A). The large number of hydroxyl, epoxy, and carboxyl groups on the surface of GO provide a negative zeta potential (ζ = − 44 mV) at a low BAC_12_ concentration of 0.0003%, and the GO solution forms a stable dispersion (Fig. 3B, left). In contrast, charge reversal was observed with increasing BAC_12_ concentration, and the ζ-potential depended on the BAC_12_ concentration. At a concentration of 0.03%, a value slightly above zero value (ζ = 3 mV) was obtained (i.e., isoelectric point) because positively charged BAC_12_ was adsorbed on the negatively charged GO surface. This neutralizing charge caused GO aggregation in water, and GO floating on the water surface (i.e., non-wetting of GO by water, as shown in Fig. 3B, right) indicated a hydrophobic surface of BAC_12_/GO composites. The adsorbed BAC_12_ likely formed a horizontal monolayer on the GO surface, as reported for cationic surfactant/graphite surfaces^18^. The hydrocarbon chains of BAC_12_ face the water phase and increase the hydrophobicity of the GO surface. As the concentration of BAC_12_ was further increased, the ζ-potential increased from + 23 mV at 0.1 wt% to + 53 mV at 1.7 wt%, indicating that more BAC_12_ molecules were bound to the hydrophobic GO surface to form hemicylindrical aggregates^19^. We also found that some of the BAC_12_ surfactant was tightly adsorbed by the GO surface and did not desorb, even after repeated water rinsing with six cycles of centrifugation/redispersion for a GO dispersion with 0.1 wt% BAC_12_. After this rinsing treatment, the ζ-potential for the GO dispersion was slightly positive (about 3–10 mV), which led to sedimentation of GO in water (Fig. 3C). This indicates that the BAC_12_ surfactant remains on the GO surface after the water rinsing process.

The hydrophilicity of each quartz substrate is shown in Fig. 3D. The contact angles for untreated, BAC, CPC, and BZC-treated substrates were 48.4°, 56.7°, 53.7°, and 58.4°, respectively. In contrast, the GO-treated substrate was highly hydrophilic, with a contact angle of 12.9°. The hydrophilicity of GO due to the large number of oxygen functional groups has been reported previously^20^. These results suggest that GO remains on the quartz substrate after the water spraying process. In contrast, the contact angles for GO/BAC, GO/CPC, and GO/BZC were 51.6°, 46.0°, and 41.8°, respectively, showing increased hydrophobicity compared to the substrate treated with GO alone. This suggests that the CSAA bound to the surface of the GO film. The same trend was observed for the PET (Supplementary Fig. 2) and polystyrene (Supplementary Fig. 3) substrates, suggesting that GO/CSAA films can be formed on a variety of substrates. The water storage tests using quartz substrates in water for 7 and 14 days showed that GO/CSAA tended to remain on hydrophobic surfaces, especially GO/BAC, compared to substrates without GO.

### Antibacterial effect of GO/CSAA ultrathin film

**Figure 4:**
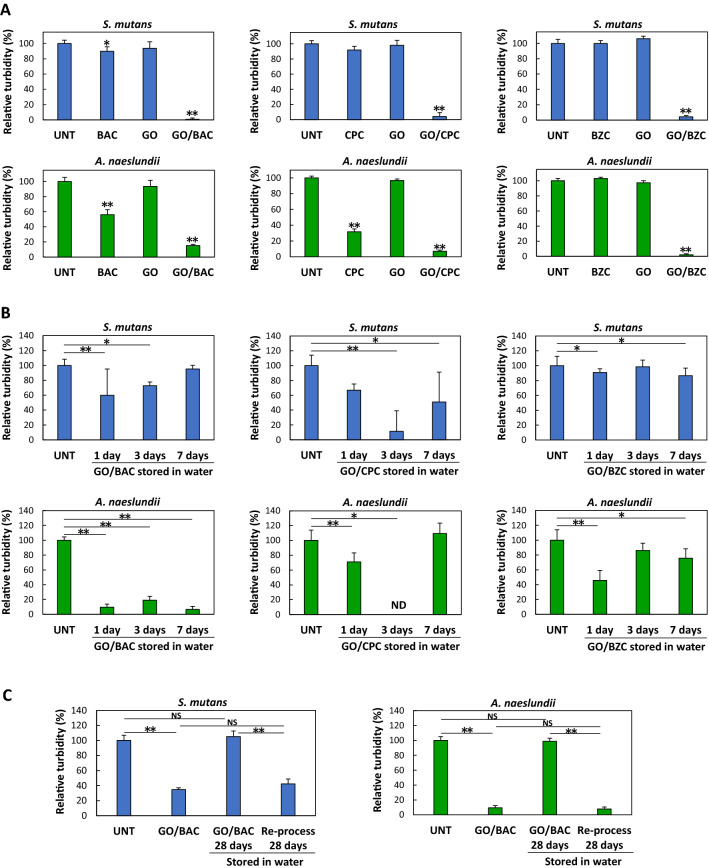
Antibacterial assessment of GO/CSAA. (**A**) Relative turbidity (n = 6, mean + standard deviation) after 24 h incubation of *S. mutans* (top) and *A. naeslundii* (bottom) cultured on each well substrate. *P < 0.05 vs. UNT. **P < 0.01 vs. all other groups. (**B**) Long-term durability of GO/CSAA under water storage. Relative turbidity (n = 6, mean + standard deviation) of *S. mutans* (top) and *A. naeslundii* (bottom) cultured on each well substrate. *P < 0.05, **P < 0.01. (**C)** Antibacterial assessments after reprocessing with BAC. Relative turbidity (n = 6, mean + standard deviation) of *S. mutans* (left) and *A. naeslundii* (right) cultured on each well substrate. **P < 0.01. Statistical analysis: two-tailed one-way ANOVA with post-hoc Tukey HSD test. *BAC* benzalkonium chloride, *BZC* benzethonium chloride, *CPC* cetylpyridinium chloride, *CSAA* cationic surface active agent, *GO* graphene oxide, *ND* not detected, *NS* not significant, *UNT* untreated.

The antibacterial properties of GO/CSAA were assessed by incubation with bacterial cells for 24 h and the results are shown in Fig. 4A. The relative turbidity due to *S. mutans* and *A. naeslundii* was similar in all of the GO-treated wells and not significantly different from that in untreated wells, showing that the GO ultrathin film alone has no antibacterial properties. GO is reported to exhibit antimicrobial activity due to the “nanoknife” edge effect of GO nanosheets, or to the induction of reactive oxygen species, both of which damage the cell membrane^21^. However, high concentrations of GO are required for strong antimicrobial activity^22,23^. In the present study, GO nanosheets comprised a single layer adhered to the substrate and were not dispersed in the medium. In addition, excess GO was removed by rinsing the GO film with water, suggesting that the remaining amount of GO was small relative to the total amount of bacteria, likely leading to a lack of antibacterial activity. In some cases, CSAA treatment alone, especially with BAC and CPC, resulted in antibacterial activity compared to the untreated control (Fig. 4A). As shown in Supplementary Fig. 3, following treatment of the surface of the tissue culture dish with CSAA, the contact angle was increased slightly compared to untreated culture dishes, suggesting that trace amounts of CSAA bound to the surface and exerted bacterial inactivation.

In contrast, all of the GO/CSAA-treated wells showed significantly lower relative turbidities than the control (P < 0.01) (Fig. 4A), suggesting that the growth of bacterial cells was inhibited and that GO/CSAA may be a revolutionary coating that exhibits antibacterial activity even in wet conditions. Fujii et al. fabricated aggregates of GO and CSAA to demonstrate the release of CSAA from GO^24^. They reported that the antimicrobial effect of GO/CSAA requires GO reduction and subsequent CSAA release. CSAA retained on GO ultrathin films will likely be gradually released and exhibit antimicrobial activity in water (the contact angle test in Fig. 3D also showed that hydrophobicity of GO/CSAA was lost in a time-dependent manner). However, since complete loss of CSAA during extended storage in water would decrease the effectiveness of the coating, we investigated whether the antibacterial properties of GO/CSAA were maintained after long-term storage in water by subsequent seeding with bacterial suspensions.

Antibacterial testing was conducted using GO/CSAA stored in water for up to 7 days (Fig. 4B). The turbidity of GO/CSAA-treated wells was significantly suppressed compared to untreated wells, suggesting that CSAA adhered to the GO ultrathin film even after long-term storage in water. Thus, the GO film appeared to inhibit the immediate diffusion of CSAA in water and may be useful as a long-term antimicrobial coating in wet conditions. In contrast, even though GO/BAC was the most hydrophobic composite, based on the results of the contact angle measurements (Fig. 3D), the turbidity of GO/BAC did not decrease and was comparable to that of the untreated well after storage in water for 28 days (Fig. 4C). These findings suggest that CSAA is released over time during storage in water and was almost entirely released after one month when no antibacterial activity was observed. This GO/BAC lacking antibacterial activity after 28 days was reprocessed with 0.1% BAC. When incubated with *S. mutans* or *A. naeslundii,* a decrease in turbidity comparable to that of GO/BAC not stored in water was observed. Thus, CSAA retained on the surface may be released in a time-dependent manner, while GO films remained on the substrate surface after storage in water and could rebind with the CSAA.

### Water resistance and antibacterial mechanism of GO/CSAA

**Figure 5:**
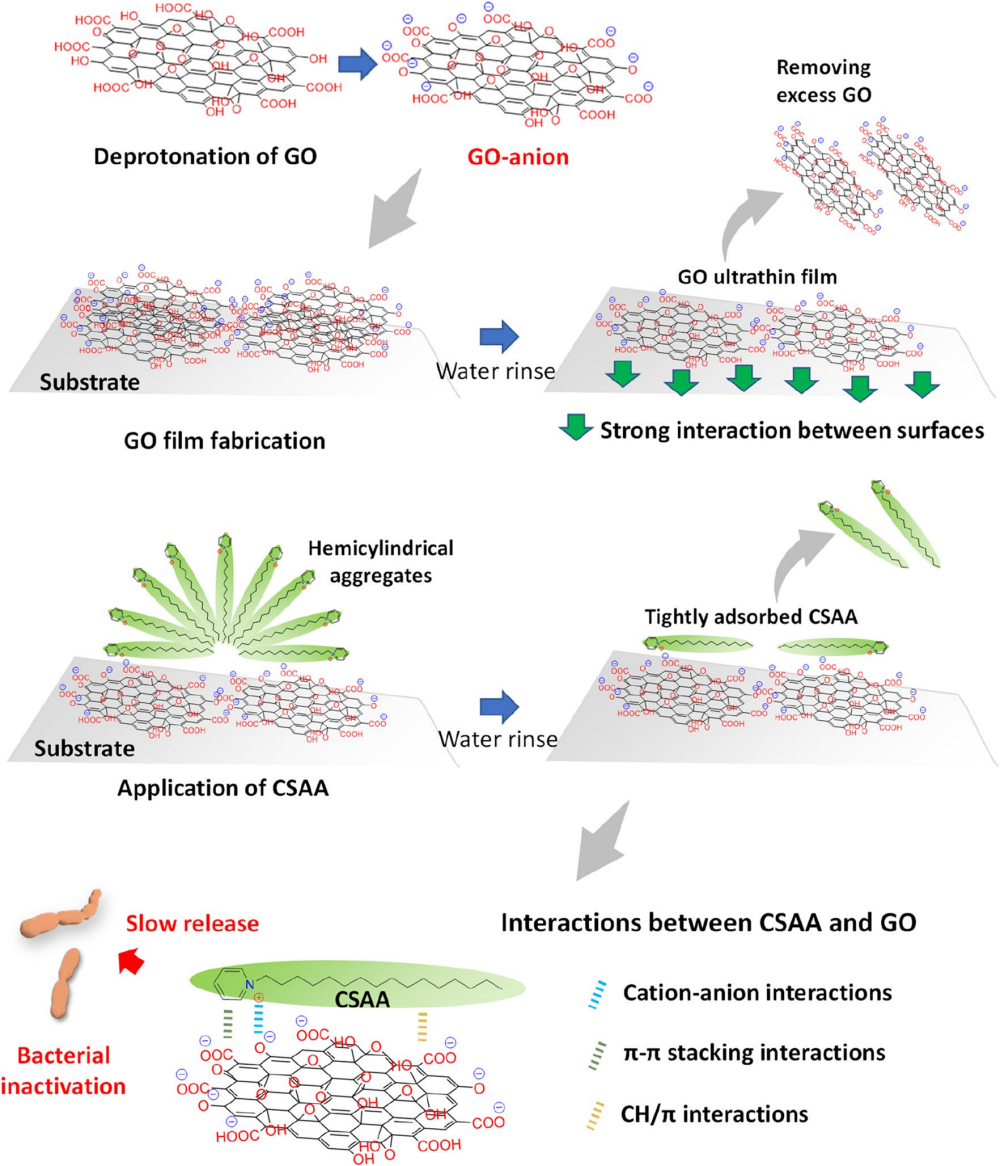
Schematic representation of the mechanism of water resistance and antibacterial action of GO/CSAA. *CSAA* cationic surface active agent, *GO* graphene oxide.

The proposed mechanism underlying the water resistance and antibacterial properties of GO/CSAA is shown schematically in Fig. 5. GO has a sheet-like structure and adheres well to the substrate due to high surface-to-surface interaction (e.g., van der Waals forces and ionic interactions). After GO film fabrication by drying, excess GO is removed from the substrate by water rinsing but the single layer of GO closest to the substrate is retained by strong attractive forces. GO is a highly anionic substance and strongly interacts with a cationic substrate. In particular, the tooth substrate, the target of this study, is composed mainly of hydroxyapatite and thus is rich in cationic calcium ions^25^, indicating that teeth have excellent affinity for GO.

The CSAA strongly binds the GO surface by anion-cation interactions, which prevent its removal even after the water rinsing process. In addition, synergistic effects from other effects such as π-π stacking and CH/π interactions^26–29^ likely further prevent CSAA removal. Previous studies examined the adsorption properties of anionic and cationic dyes for GO and reduced GO and showed that GO strongly adsorbs cationic dyes through electrostatic interactions, whereas reduced GO (with fewer anionic functional groups) adsorbs both anions and cations through charge-independent interactions (e.g., presumably through stacking interactions and/or van der Waals forces)^30,31^.

Interestingly, we observed that CSAA was released from GO/CSAA to provide long-term antibacterial activity in water, and that CSAA could be reapplied to the depleted GO substrate, reconferring an antibacterial effect. We propose the following hypothesis to explain why CSAA is released and can rebind to a GO film through water storage. Desorption of CSAA from GO may occur by ion exchange in the presence of an excess amount of water or CO_2_ from the air (H^+^, OH^-^ and HCO_3_^-^ ions), as suggested by a previous study^24^, exposing anionic functional groups remaining on the GO surface that are receptive to CSAA rebinding upon applying an excess amount of CSAA.

**Figure 6:**
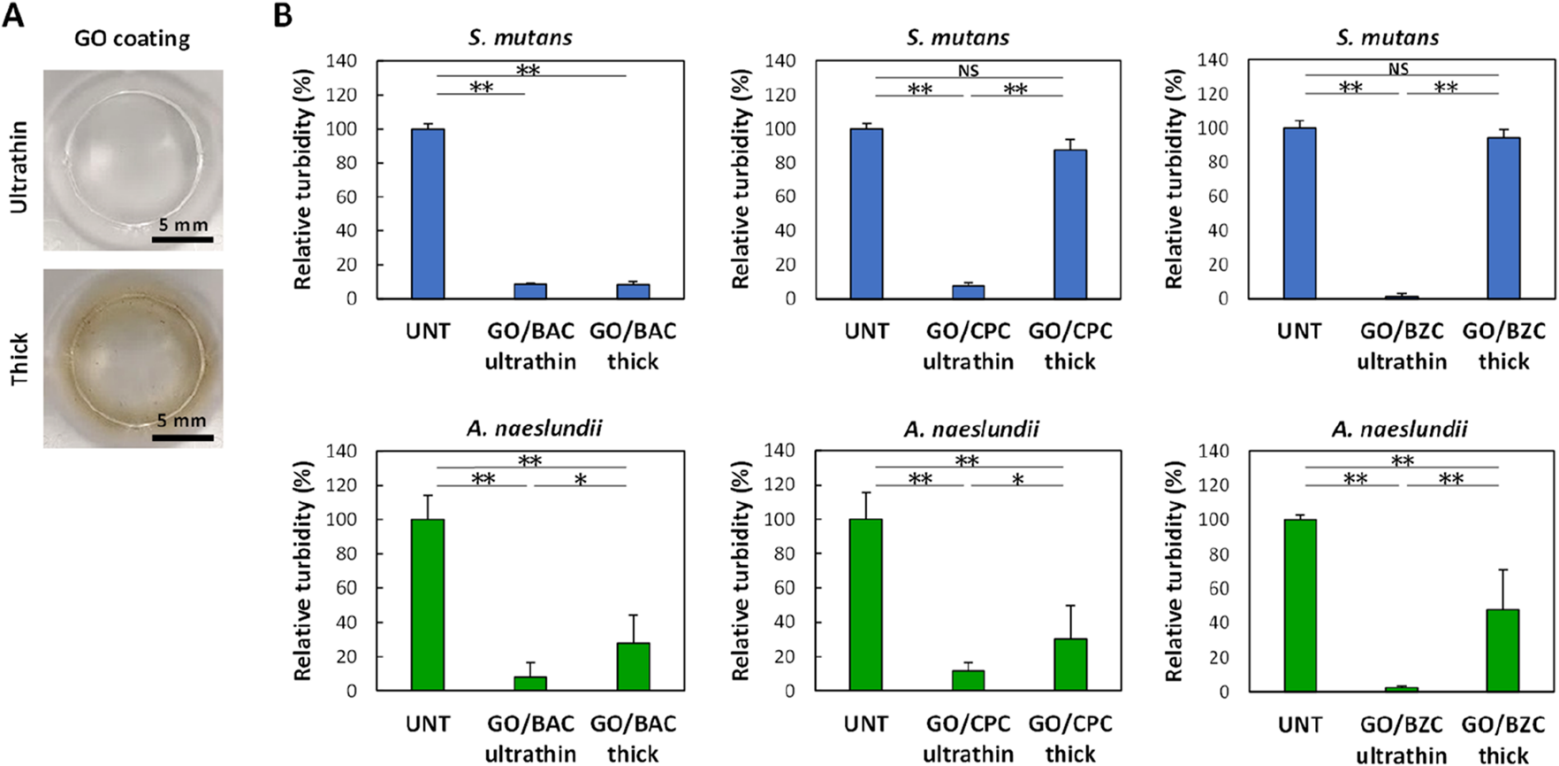
Comparison of GO ultrathin and thick films. (**A**) Digital photographs of culture well coated with GO ultrathin and thick films. (**B**) Relative turbidity (n = 6, mean + standard deviation) of *S. mutans* (top) and *A. naeslundii* (bottom) cultured on each well substrate. *P < 0.05, **P < 0.01. Statistical analysis: two-tailed one-way ANOVA with post-hoc Tukey HSD test. *BAC* benzalkonium chloride, *BZC* benzethonium chloride, *CPC* cetylpyridinium chloride, *GO* graphene oxide, *NS* not significant, *UNT* untreated.

A GO ultrathin film coating is advantageous as it does not change the color of the substrate. In addition, the strong interaction between GO and CSAA in a thick GO film used as an antibacterial coating could cause the release of CSAA due to interlayer delamination of the GO film. We therefore prepared GO ultrathin films and GO thick films (not rinsed with water) (Fig. 6A), which were then complexed with the CSAA to compare their antibacterial activity. Although some of the GO thick films exhibited antibacterial activity, the GO ultrathin films were superior in consistently inhibiting turbidity (Fig. 6B).

### Antibacterial effect of GO/CPC on human tooth substrates

**Figure 7:**
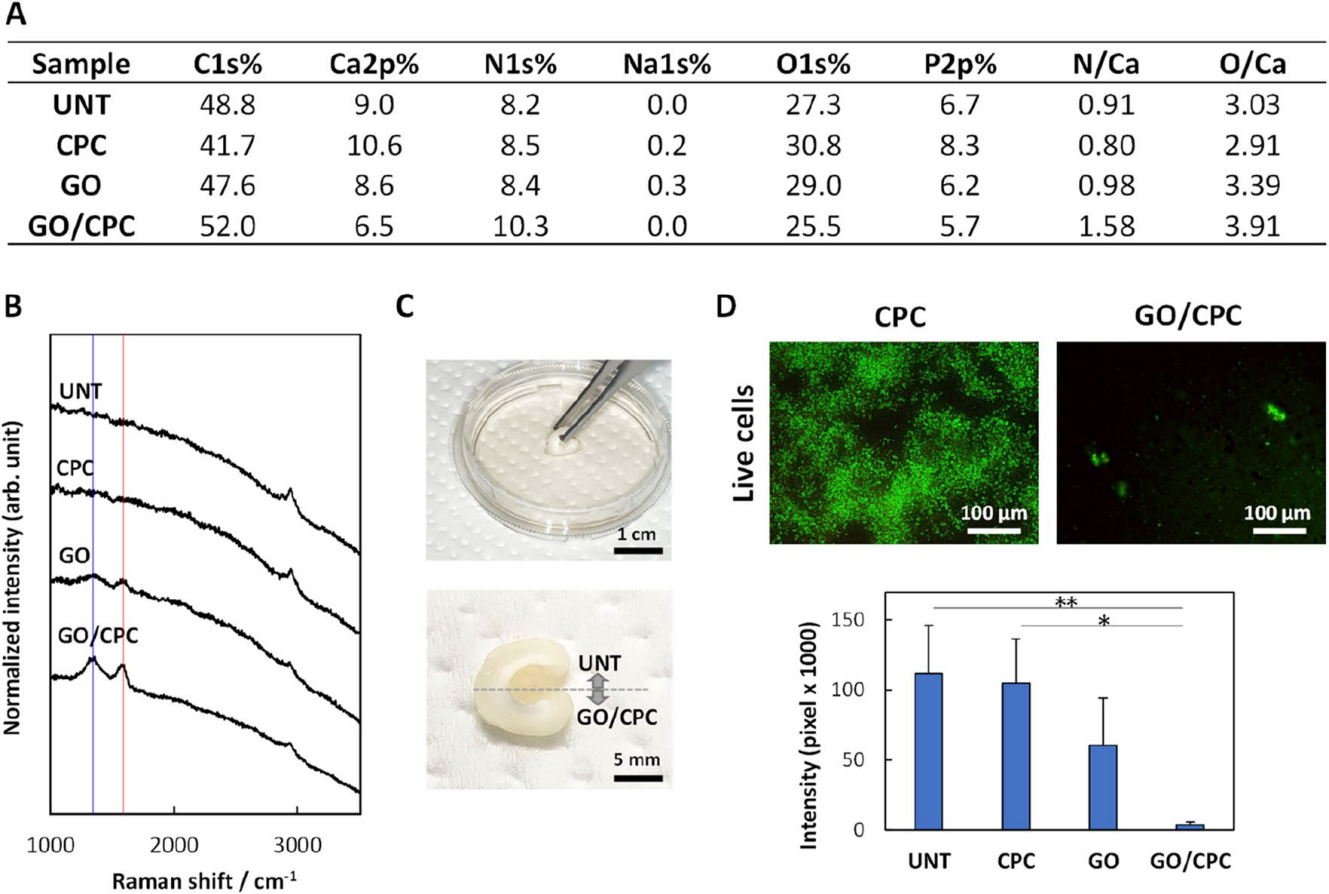
Characterization and antibacterial activity of GO/CPC on human tooth substrate. (**A**) XPS elemental analysis. (**B**) Raman spectroscopy analyses. The red and blue lines indicate 1600 cm^-1^ and 1350 cm^-1^ (G and D bands), respectively. (**C**) Digital images of tooth substrate coated with GO/CPC. Only the lower half of the tooth substrate was immersed in 0.01% GO dispersion (top). No color change was observed of the GO/CPC coated area compared to UNT (bottom). (**D**) Fluorescent staining (top; green indicates live *S. mutans*) and quantification of the staining intensity of *S. mutans* cultured on each tooth substrate (bottom; n = 3, mean + standard deviation). *P < 0.05, **P < 0.01. Statistical analysis: two-tailed one-way ANOVA with post-hoc Tukey HSD test. *CPC* cetylpyridinium chloride, *GO* graphene oxide, *UNT* untreated.

Figure 7A shows the results of an XPS elemental analysis of variously treated tooth substrates: untreated, CPC alone, GO alone, and GO/CPC. The O1s and N1s peaks were higher for GO/CPC-treated tooth substrates than for the other treatments. However, dentin, the substrate used in this study, is an organic–inorganic composite of calcium phosphate deposited on a collagen network and is naturally rich in O and N^32^. Therefore, we compared the ratio of O or N with respect to Ca, to verify the presence of the GO/CPC ultrathin film. The results showed that both the N/Ca and O/Ca ratios for GO/CPC were larger than those for other treated substrates, suggesting the presence of CPC and GO, respectively. In addition, the Raman spectra (Fig. 7B) showed G and D bands (red and blue lines in Fig. 7B, respectively) for the GO-treated and GO/CPC-treated tooth substrates. These results indicate that GO or GO/CPC remained on the tooth surface even after rinsing in water, similar to the results obtained using quartz substrates. To determine if there was any color change of the tooth substrate, the lower half of the sample was immersed in a 0.01% GO dispersion and a CPC agent. We observed that the GO/CPC area was comparable in color to the untreated area (Fig. 7C).

The antibacterial effect of each treated tooth substrate is shown in Fig. 7D. Fluorescent staining indicated that CPC treatment alone supported the formation of bacterial biofilms on the tooth surface (colored in green), whereas GO/CPC showed no biofilm and few bacterial cells. Quantification of the fluorescent area using biofilm images of each substrate showed that the CPC-treated substrate exhibited the same fluorescence intensity as the untreated tooth, suggesting that even though the tooth surface was coated with CPC, the CPC was quickly removed by water and a biofilm formed. In contrast, the GO/CPC-treated substrate showed significantly lower fluorescence intensity than the other groups (P < 0.05), suggesting that GO/CPC inhibits the growth of bacterial cells. Thus, the combination of CPC and GO ultrathin film is likely a clinically significant method for addressing dental disease. The silver diamine fluoride coating method (silver deposited on the tooth surface) is currently used to prevent dental caries, but the silver coating causes darkening of the teeth, which is problematic from an esthetic standpoint^33^. GO/CSAA is transparent and does not alter the color of teeth, so satisfying esthetic requirements. Further clinical studies are needed to determine whether GO/CSAA ultrathin films can sustain antibacterial and antibiofilm activity in the wet environment of the oral cavity, and whether they can inhibit oral diseases such as dental caries and periodontitis.

### Cytocompatibility assessments of GO/BAC ultrathin films

**Figure 8:**
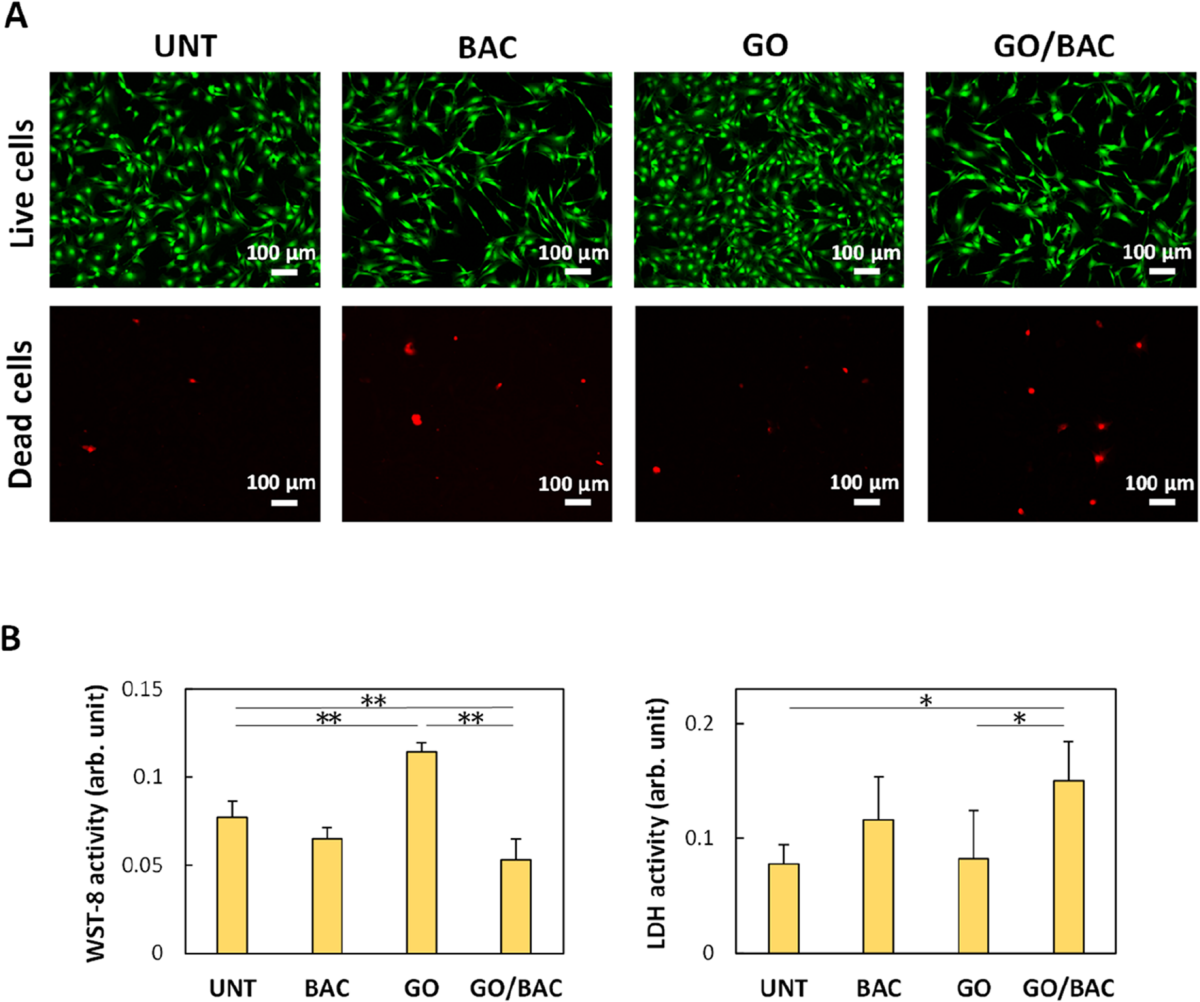
Cytocompatibility assessments of GO/BAC. (**A**) Fluorescent staining (top: green and red indicate live and dead NIH3T3 cells, respectively). (**B**) WST-8 (left) and LDH activity (right) of NIH3T3 cells cultured on each well substrate (n = 6, mean + standard deviation). *P < 0.05, **P < 0.01. Statistical analysis: two-tailed one-way ANOVA with post-hoc Tukey HSD test. *BAC* benzalkonium chloride, *GO* graphene oxide, *LDH* lactate dehydrogenase, *UNT* untreated, *WST* water-soluble tetrazolium salt.

Biocompatibility tests using antibacterial GO/BAC, the most long-term stable ultrathin film, were conducted (Fig. 8). Live (green)/dead (red) staining of mammalian cells after culturing for 24 h showed many living cells and few dead cells in each treatment well (Fig. 8A). Notably, the GO-treated wells tended to have more fluorescent cells, confirming the results of the WST-8 test (Fig. 8B, left). The WST-8 assay showed that the viability of fibroblastic cells was significantly higher on GO films than on untreated substrates (P < 0.01). This interesting result may depend on the ability of GO to adsorb substances. It has been reported that GO attracts many of the adhesion molecules necessary for cell attachment and growth in culture medium, thus providing a biological environment to selectively increase cell activity^34–36^.

Various studies have reported the cytotoxicity of GO, and the degree of cytotoxicity depends on the amount of GO. GO exhibits little toxicity to fibroblasts when the GO concentration is below 20 μg/mL^37^. Nishida et al. prepared GO films (total amount of 10 μg GO per well) on the bottom of culture wells, similar to the current research, and observed that cell proliferation was inhibited^38^. The amount of GO cast in each well in the present study was 0.1 μg. Furthermore, much of the GO film was removed by the water spraying process, so the amount remaining in the wells was likely extremely small. The lower WST-8 value and higher LDH activity (cytotoxicity) of GO/BAC compared to the untreated control may be due to the effect of combined BAC, rather than GO toxicity (Fig. 8B). GO/BAC exhibited a weak effect towards mammalian cells compared to its strong action on oral bacteria, thus removing one concern for its clinical application.

## Conclusion

In this study, we developed a new coating method using GO and a CSAA as a water-resistant antimicrobial coating. A transparent ultrathin film of GO (almost a monolayer) was formed on the surface of the substrate and then composited with a CSAA. The formation of GO/CSAA was confirmed by SEM, Raman, XPS, ζ-potential and contact-angle analyses, and we confirmed that GO and CSAA were not removed even after rinsing with water. GO/CSAA inhibited the growth of *S. mutans* and *A. naeslundii*. Although GO/CSAA maintained its antibacterial activity after storage in water, no antibacterial activity was observed after long-term storage in water (28 days). However, re-addition of the CSAA recovered the antibacterial effect of GO/CSAA. These results suggest that GO/CSAA has water-resistant properties (suitable for repeated use) in addition to antibacterial activity. Furthermore, GO/CSAA formed easily on human tooth substrates and showed antibacterial effects. In addition, GO ultrathin films were cytocompatible with mammalian cells. Our findings show that GO/CSAA exhibited a sustained antibacterial effect in a wet environment and might be beneficial in the treatment and prevention of oral infectious diseases. Specifically, the use of a mouthrinse containing a CSAA every few days after coating the teeth with GO may provide the teeth with long-term antibacterial properties. In addition, regular coating of teeth with GO could be a low-maintenance therapy for oral antimicrobial protection.

## Supplementary Information

Supplementary Figures.

## Data Availability

The datasets are available from the corresponding author upon reasonable request.
