# Supplementary material for: Sustained antibacterial coating with graphene oxide ultrathin film combined with cationic surface-active agents in a wet environment
Source: Sci Rep. 2022 Oct 18;12:16721. doi: 10.1038/s41598-022-21205-4 (PMC9579177; doi:10.1038/s41598-022-21205-4)
Supplement: Supplementary file 1 — Supplementary Figures. [file 41598_2022_21205_MOESM1_ESM.docx]

**Sustained antibacterial coating with graphene oxide ultrathin film combined with cationic surface-active agents in a wet environment**

Hirofumi Miyaji^1*^, Yukimi Kanemoto^1^, Asako Hamamoto^1^, Kanako Shitomi^2^, Erika Nishida^1^, Akihito Kato^1^, Tsutomu Sugaya^1^, Saori Tanaka^1,3^, Natsuha Aikawa^4^, Hideya Kawasaki^4^, Syun Gohda^5*^, Hironobu Ono^5^

**Affiliations**

^1^ Department of Periodontology and Endodontology, Faculty of Dental Medicine, Hokkaido University, N13W7, Kita-ku, Sapporo, Hokkaido 060-8586, Japan

^2^ Division of Periodontology and Endodontology, Department of Oral Rehabilitation School of Dentistry, Health Sciences University of Hokkaido, 1757 Kanazawa, Tobetsu-cho, Ishikari-gun, Hokkaido 061-0293, Japan

^3^ Division of General Dentistry Center for Dental Clinics, Hokkaido University Hospital, N14W5, Kita-ku, Sapporo, Hokkaido 060-8648, Japan

^4^ Department of Chemistry and Materials Engineering, Faculty of Chemistry, Materials and Bioengineering, Kansai University, 3-3-35 Yamate-cho, Suita, Osaka 564-8689, Japan

^5^ Nippon Shokubai Co., Ltd, 5-8 Nishiotabi-cho, Suita, Osaka 564-0034, Japan

**Corresponding authors**

Hirofumi MIYAJI

Department of Periodontology and Endodontology, Faculty of Dental Medicine, Hokkaido University, N13, W7, Kita-ku, Sapporo, Hokkaido 060-8586, Japan.

E-mail: [miyaji@den.hokudai.ac.jp](mailto:miyaji@den.hokudai.ac.jp)

Syun GOHDA

Nippon Shokubai Co., Ltd, 5-8 Nishiotabi-cho, Suita, Osaka 564-0034, Japan.

E-mail: [shun_goda@shokubai.co.jp](mailto:shun_goda@shokubai.co.jp)

**Supplementary Figure 1. XPS spectral analysis of Cl2p and O1s.**

Abbreviations: BAC, benzalkonium chloride; BZC, benzethonium chloride; CPC, cetylpyridinium chloride; GO, graphene oxide; XPS, X-ray photoelectron spectroscopy; UNT, untreated.

**Supplementary Figure 2. Hydrophilicity of GO/CSAA on PET substrate.**

The number above each droplet is the contact angle obtained for the sample.

Abbreviations: BAC, benzalkonium chloride; GO, graphene oxide; PET, polyethylene terephthalate; UNT, untreated.

**Supplementary Figure 3. Hydrophilicity of GO/CSAA on** **polystyrene substrate.**

The number above each droplet is the contact angle obtained for the sample.

Abbreviations: BAC, benzalkonium chloride; BZC, benzethonium chloride; CPC, cetylpyridinium chloride; CSAA, cationic surface active agent; GO, graphene oxide; UNT, untreated.
